# Comorbidities associated with the severity of COVID-19, and differences across ethnic groups: a UK Biobank cohort study

**DOI:** 10.1186/s12889-023-16499-6

**Published:** 2023-08-17

**Authors:** Rahul Patel, Jaspal S. Kooner, Weihua Zhang

**Affiliations:** 1 Department of Epidemiology and Biostatistics, Imperial College London, London, W2 1PG UK; 2 Guy’s, King’s and St Thomas’ School of Medical Education, King’s College London, London, SE1 1UL UK; 3 Department of Cardiology, Ealing Hospital, London North West University Healthcare NHS Trust, London, UB1 3HW UK; 4 National Heart and Lung Institute, Imperial College London, London, W12 0NN UK; 5 Imperial College Healthcare NHS Trust, London, W12 0HS UK; 6 MRC-PHE Centre for Environment and Health, Imperial College London, London, W2 1PG UK

**Keywords:** COVID-19, Comorbidities, UK Biobank, Cohort, Ethnicity, Disparity

## Abstract

**Background:**

Disparities in COVID-19 outcomes exist on the basis of ethnicity and comorbidities. Minority ethnic groups in the UK are known to have poorer COVID-19 outcomes, but also an increased prevelance of certain comorbidities associated with severe outcomes. Additionally, despite the prevalence of certain psychiatric disorders there is a lack of research establishing their relationship with COVID-19 outcomes.

**Methods:**

We used UK Biobank data, involving 472,182 participants, to test for an association between comorbidities and COVID-19 diagnosis (*n* = 30,901); and to test for an association between comorbidities and severe COVID-19 (*n* = 3182). This was done by performing univariable and multivariable logistic regression analysis, estimating odds ratios (ORs) and their 95% confidence intervals (95% CIs). The comorbidities studied were coronary heart disease (CHD), hypertension, type II diabetes mellitus (T2DM), obesity, chronic kidney disease (CKD), depression and anxiety. Multivariable models were adjusted for various socioeconomic, demographic and health-related confounders. We then performed sub-group analysis by common UK ethnic groups (White, South Asian, and Black).

**Results:**

Increased prevalence of all studied comorbidities was seen in both outcomes, compared to the rest of the cohort. All studied comorbidities were associated with an increased risk of COVID-19 infection and severity across all models. For example, the adjusted ORs (95% CI) for depression were 1.112 (1.083 – 1.161) for COVID-19 diagnosis and 2.398 (2.163 – 2.658) for severe COVID-19. Sub-group analysis revealed stronger associations of COVID-19 diagnosis and severe COVID-19 for South-Asian participants for CHD (OR 1.585 [95% CI 1.194–2.105] for COVID-19 diagnosis and 3.021 [1.683–5.390] for severe COVID-19), hypertension (1.488 [1.231–1.799]; 3.399 [1.862–6.206]) and T2DM (1.671 [1.346–2.076]; 5.412 [3.130–9.357]) compared to White participants (1.264 [1.195–1.336] and 1.627 [1.441–1.837] for CHD; 1.131 [1.097–1.116] and 2.075 [1.885–2.284] for hypertension; 1.402 [1.331–1.476] and 2.890 [2.596–3.216] for T2DM). Similar results were seen for Black participants with CKD and hypertension.

**Conclusion:**

Specific comorbidities are risk factors for poorer COVID-19 outcomes, supporting targeted interventions and policy aimed at individuals with these comorbidities. Although further research is required, there’s also a need for targeted policies for ethnic minorities assessing the unique reasons they are at greater risk of poor COVID-19 outcomes.

**Supplementary Information:**

The online version contains supplementary material available at 10.1186/s12889-023-16499-6.

## Background

Severe acute respiratory syndrome coronavirus 2 (SARS-CoV-2) was declared a global pandemic by the World Health Organisation on 11 March 2020 due to its rapid spread and pathogenicity [1]. The first confirmed case of SARS-CoV-2 infection in the UK occurred on 30 January 2020, and as of 19 July 2023 there has been a total of 22,241,790 confirmed cases and 228,492 deaths related to the infection [2]. Clinically, SARS-CoV-2 causes the illness known as COVID-19 (coronavirus disease 2019) [3]. However, clinical manifestation of COVID-19 exists on a spectrum, which can range from being asymptomatic to critical illness, including death [3]. Although everyone is at risk of infection with SARS-COV-2 and consequential critical illness, certain groups are known to have increased risk of infection and severe disease. Disparities are known to exist on the basis of various factors including sex, age, ethnicity, occupation, deprivation, smoking status, and medical comorbidities [3, 4].

Certain comorbidities are known to be associated with severe COVID-19 outcomes, such as having a diagnosis of a certain condition can confer poor COVID-19 outcomes. Diagnoses of hypertension and type II diabetes mellitus (T2DM) have been strongly associated to poor COVID-19 outcomes indepdendently, in both global and UK-based settings [4, 5]. Moreover, chronic kidney disease (CKD), obesity and cardiovascular disease have also been associated to poorer COVID-19 outcomes [3, 4, 6]. In the context of the UK, ethnic minority groups (often referred to as BAME [Black, Asian and minority ethnic] groups) saw an increased risk of infection, need of critical care and mortality compared to White ethnic groups [4]. Health and ethnicity have a complicated association; many unique societal and cultural reasons could explain the trend seen, such as household and occupational factors [4, 7]. Notably, there is a relationship between ethnicity and prevalence of comorbidities, with certain comorbidities having a higher prevalence in BAME communities [4]. For example, T2DM, coronary heart disease (CHD) and obesity are more prevalent in certain groups within BAME communities, compared to White ethnic groups [4, 8].

Psychiatric disorders have been linked to an elevated risk of infection [9]. Therefore, diagnosis of a psychiatric disorder may be associated with COVID-19 outcomes. However, despite that certain mental health conditions are highly prevalent in the UK (1 in 6 [15.7%] of people aged over 16 experiencing symptoms of anxiety and depression) there are few studies exploring the risk of psychiatric disorders and COVID-19 risk [10]. Studies that explore this association observe an increased risk of severe COVID-19 outcomes for conditions such as depression and anxiety [11, 12].

The UK Biobank (UKB) is a UK-based long-term prospective study containing ~ 500,000 participants, aged between 40–69 at the time of recruitment (2006–2010) [13]. It contains extensive information on participants, including health-related behaviours and outcomes (e.g., smoking status), lifestyle factors, socioeconomic and demographic information [13]. It also has linked hospital records and mortality data [14]. Data was collected at base-line assessment and is being added upon regularly through longitudinal follow-up and linked data providers feeding into the system [13]. Crucially, COVID-19 data has been linked to UKB participants [15, 16].

### Research in context

There has been previous research analysing the effect of comorbidities on COVID-19 severity in the UKB cohort. We searched PubMed up to March 30, 2022, using the search terms “(COVID-19) and (comorbidit*) and (UK Biobank)”, which yielded 46 results. These studies generally found an increased risk of severe COVID-19 for individuals with certain comorbidities, such as cardiovascular disease, psychiatric disorders, gout, and Parkinson’s disease [11, 12, 17, 18]. However, due to the dynamic nature of the pandemic there is now more COVID-19 data available for the cohort, involving data up until September 2021. Few studies have utilised the larger data source now available, which this study will do thus strengthening the current evidence base. Furthermore, few studies have utilised the database to stratify their analysis by ethnicity, with a particular lack of focus on South-Asian ethnicity, despite being the largest minority group in the UK [19]. This study will include sub-group analysis by ethnicity, with dedicated ethnic groups for South-Asian and Black participants. To the best of our knowledge, it is one of the first studies to analyse the differential risk of COVID-19 outcomes that different comorbidities confer, by ethnicity. Additionally, this study will help clarify the relationship between common mental health disorders and COVID-19 outcomes.

## Methods

The primary objectives of this study are to identify which comorbidities are risk factors for COVID-19 diagnosis and severity and to identify whether a difference in comorbidities associated with diagnosis and severity is seen among different ethnic groups.

### UK Biobank

We have ethical approval through the original UKB study, which was granted by the North West Multi-centre Research Ethics Committee based in the UK. Written informed consent was obtained from all participants in the original study. This study was approved under the UKB application number 62510.

### Study design

**Fig. 1:**
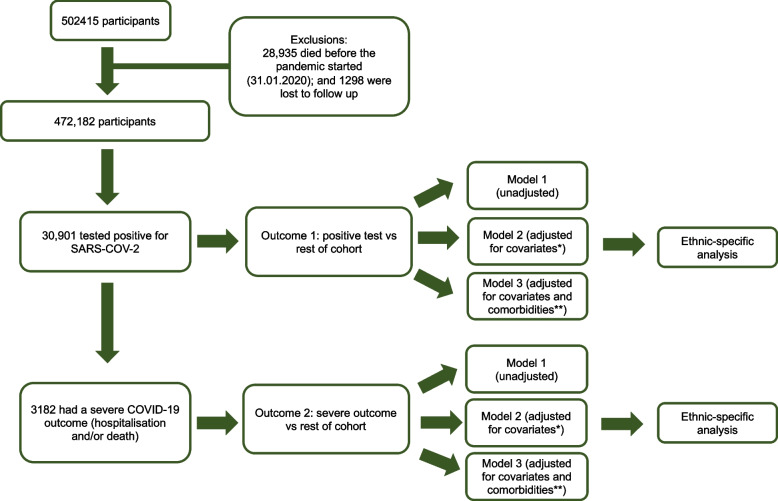
Study design flow chart, including participant numbers. *Covariates adjusted for in models 2 and 3 were ethnicity, sex, age, BMI, Townsend deprivation index, smoking status, qualifications, and pre-tax household income. **Comorbidities adjusted for in model 3 were coronary heart disease, hypertension, type II diabetes mellitus, obesity, chronic kidney disease, depression, and anxiety (BMI removed)

The UKB cohort consisted of 502,415 participants. Participants were excluded if they were lost to follow up, or if they died before the first case of COVID-19 in the UK (30 January 2020). This resulted in 472,182 participants for analysis (Fig. 1).

COVID-19 data was extracted from linked data to UKB. Data on COVID-19 reverse-transcriptase polymerase chain reaction (RT-PCR) test date, origin and results were available for England, Scotland and Wales through Public Health England (PHE), Public Health Scotland, and Secure Anonymised Information Linkage (SAIL) databank respectively. COVID-19 RT-PCR data includes testing up until September 2021. Moreover, UKB data included COVID-19 inpatient hospital data and death records coded as per the International Classification of Diseases 10^th^ revision (ICD-10). This data was available through linkages to Hospital Episode Statistic and Death Registry from NHS Digital up until September 2021 [14, 15]. We combined COVID-19 PCR test results, inpatient data, death data and ICD-10 codes (U071 or U072) to confirm those with a COVID-19 diagnosis. Severe form of COVID-19 was defined as having a hospital diagnosis with COVID-19 or died from COVID-19 from the Death Registry as a primary diagnosis based on ICD-10. We removed secondary contributing diagnoses of COVID-19 from the severe outcome, so that only those who were admitted due to a COVID-19 diagnosis were included. This was to ensure only those with a severe reaction (needing hospital admission) were included within the variable.

Comorbidity data was extracted from both baseline self-reported data (UKB Unique Data Identifier [UDI] 20,002) and hospital episode records. Baseline data was collected from UKB assessment centres at recruitment. It involved a self-questionnaire and a verbal interview with a medical professional to confirm self-reported illness. These were combined with hospital episode record data, using the ICD-10 coding. Therefore, we could ascertain the total levels of each comorbidity studied, as when self-reported illness was positive and/or hospital record data was positive we counted this as a positive comorbidity data point. The following diagnoses were looked at based on ICD-10: hypertension (I10-I15), CHD/ischaemic heart disease (I21-I23), T2DM (IE11), CKD (N18), obesity (E66), depression (F32-33) and anxiety (F41). Participants with multiple diagnoses were counted for each comorbidity they had. For obesity, we also combined the hospital episode record data with the baseline Body Mass Index (BMI). This was calculated from weight and height measurements, kg/m^2^, with > 30 kg/m^2^ indicating obesity.

Ethnicity data from UKB was self-reported from the baseline questionnaire. It was recoded into the following categories: White (combined from White British, White Irish and White other), South Asian (combined from Indian, Pakistani or Bangladeshi), Black (combined from Black African, Black Caribbean and any other black background), and other (combined from Chinese, Mixed, Asian or Asian British, any other Asian background and other ethnic group). Various socioeconomic, demographic and health related data were extracted for each participant, through the original assessment from UKB. Demographic data included sex and age; age was calculated from date of birth to the beginning of 2020 when the first case of SARS-COV-2 was confirmed. Socioeconomic data included the Townsend deprivation index, pre-tax household income and educational attainment level. The Townsend deprivation index is an area-based measure of socioeconomic deprivation, accounting for unemployment, home ownership, overcrowded housing, and car ownership [20]. UKB assigned the index score from the participants postcode, allocating this postcode to the deprivation score from the previous census. Health-related data involved smoking status and BMI.

### Statistical analysis

Initially we performed a descriptive analysis, where we analysed the proportion of comorbidities and covariates within the outcomes and by ethnicity. We ran chi-squared tests to assess the significance between proportions within COVID-19 diagnosis and within COVID-19 severe vairables. We ran unpaired t-tests to compare means within the same categories. We also reported the prevalence of comorbidities within the different ethnic groups (Supplementary Table 1, Additional File 1).

We used logistic regression models, both univariable and multivariable, to estimate the associations of different comorbidities on the risk of COVID-19 diagnosis and severe COVID-19. Odds ratios (OR) and 95% confidence intervals (CI) were calculated, with a *p* value less than 5% (*p* < 0.05) considered statistically significant. We tested two outcomes: firstly COVID-19 diagnosis against the rest of the cohort and secondly severe COVID-19 outcome against the rest of the cohort. Logistic regression models were used as it best suited to our data. This is because we wanted to predict a binary outcome – either having a COVID-19 diagnosis or not; or having a severe COVID-19 outcome or not. We had 3 models for each outcome. Model 1 was unadjusted. Model 2 was adjusted for factors known to influence COVID-19 outcomes including demographic factors (age, ethnicity, and sex), socioeconomic factors (Townsend deprivation index, pre-tax household income, and educational attainment) and health-related factors (BMI, smoking status). As multiple comorbidities often occur in one person, in Model 3, we additionally adjusted for the other comorbidities studied in addition to the covariates based on Model 2. However, we removed BMI as a covariate from all analyses in Model 3 and analyses involving obesity in Model 2, due to the collinearity between obesity and BMI (Supplementary Tables 2 and 3, Additional File 1).

We then performed subgroup analyses by ethnic group for model 2 – this involved only White, South Asian, and Black ethnicity as these are the largest ethnic groups in the UK and within the UKB cohort. The “other” category was excluded due to a mixture of ethnicities meaning no general conclusions could be made, and the small sample size for the individual groups that made up the category. To test for significant difference between the Odds Ratios of different ethnicities (comparing minority groups to the white ethnic groups) we used Z-score tests to calculate the *p* value for the difference.

Any missing values were not included in the analyses; when missing values occurred the model removed the values from the analysis.

We tested for multicollinearity between the covariates included in the analyses (Supplementary Tables 2 and 3, Additional File 1).

All analyses were carried out using IBM SPSS Statistics premium 27.

## Results

There were 502,415 from UKB participants, among whom 28,935 participants (5.8%) died before 30/01/2022 and were removed from the dataset. Furthermore, 1,298 participants were lost to follow up or withdrawn their consent for use by UKB. This resulted in 472,182 participants included in our analysis (Fig. 1). Of the 472,182 participants, 30,901 (6.5%) had confirmed diagnosis of COVID-19 and 3,182 (0.7%) had a severe COVID-19 outcome. Participant’s characteristics were stratified by confirmed diagnosis of COVID-19 and by a severe COVID-19 outcome (Table 1).

**Table 1:** Characteristics of the UK Biobank cohort, stratified by outcomes of COVID-19 diagnosis or severe COVID-19 outcome

		COVID-19 Diagnosis (*N* = 30,901)	Rest of Cohort (*N* = 441,281)	Severe COVID-19 Outcome (*N* = 3182)	Rest of Cohort (*N* = 469,000)
Ethnicity	White	28,295 (92%)	415,428 (95%)	2846 (90%)	440,887 (95%)
	South Asian	910 (3%)	6749 (2%)	95 (3%)	7564 (2%)
	Black	724 (2%)	7020 (2%)	116 (4%)	7628 (2%)
	Other (Including Mixed and Chinese)	818 (3%)	9685 (2%)	97 (3%)	10,406 (2%)
Sex	Female	16,260 (53%)	244,708 (55%)	1327 (42%)	259,641 (55%)
	Male	14,641 (47%)	196,572 (45%)	1855 (58%)	209,358 (45%)
Age (years)		**65 (9)**	**68 (8)**	**71 (8)**	**68 (8)**
Body Mass Index (Kg/m^2^)		**28.11 (5.02)**	**27.33 (4.74)**	**29.79 (5.75)**	**27.37 (4.75)**
Townsend Deprivation Index^a^		**-0.89 (3.20)**	**-1.36 (3.06)**	**-0.01 (3.48)**	**-1.34 (3.07)**
Smoking Status	Current	3365 (11%)	43,727 (10%)	494 (16%)	46,598 (10%)
	Previous	10,804 (35%)	149,665 (34%)	1321 (42%)	159,148 (34%)
	Never	16,556 (54%)	245,402 (56%)	1331 (42%)	260,627 (56%)
Qualifications	Degree	8276 (27%)	145,645 (34%)	618 (20%)	153,303 (33%)
	School-leaver	12,651 (42%)	165,387 (38%)	995 (32%)	177,043 (38%)
	Professional Qualification	3770 (12%)	50,744 (12%)	451 (15%)	54,063 (12%)
	None of the above	5539 (18%)	70,947 (16%)	1019 (33%)	75,467 (16%)
Pre-tax Household Income (British £)	< 18,000	5809 (22%)	81,645 (22%)	1047 (42%)	86,407 (22%)
	18,000 – 39,999	6361 (24%)	94,965 (25%)	613 (25%)	100,713 (25%)
	31,000 – 51,999	7391 (28%)	98,788 (26%)	481 (19%)	105,698 (27%)
	52,000 – 100,000	5571 (21%)	78,080 (21%)	275 (11%)	83,376 (21%)
	> 100,000	1372 (5%)	20,874 (6%)	57 (2%)	22,189 (6%)
Coronary Heart Disease Diagnosis		2076 (7%)	25,500 (6%)	519 (16%)	27,057 (6%)
Hypertension Diagnosis		11,549 (37%)	158,911 (36%)	2153 (68%)	168,307 (36%)
Type II Diabetes Mellitus Diagnosis		3013 (10%)	29,263 (7%)	891 (28%)	313,385 (7%)
Obesity		9502 (31%)	109,782 (25%)	1456 (46%)	117,828 (25%)
Chronic Kidney Disease Diagnosis		1650 (5%)	15,684 (4%)	611 (19%)	16,723 (4%)
Depression Diagnosis		3456 (11%)	38,719 (9%)	668 (21%)	41,507 (9%)
Anxiety Diagnosis		1944 (6%)	21,137 (5%)	447 (14%)	22,634 (5%)

Data are presented as N (%) or **mean (standard deviation)**

All *P* values < 0.001 for chi-squared (n) or unpaired t-test (mean)

^a^A higher Townsend deprivation index indicates a higher level of deprivation

The mean age at the start of the COVID-19 outbreak for those with a COVID-19 diagnosis (65 years) was lower than that of the rest of the cohort (68 years), whereas those with severe COVID-19 had a higher mean age (71 years) compared to the rest of the cohort. Across all outcomes the population consisted mainly of those who identified as White (ranging from 90–95%). South Asian participants saw an increased representation in the number of positive cases and severe outcomes (910 [3%] diagnoses vs. 6749 [2%] rest of the cohort and 95 [3%] severe outcome vs. 7564 [2%] rest of cohort). Black participants saw an increased proportion in severe cases (116 [4%] severe outcome vs. 7628 [2%] rest of the cohort).

**Fig. 2:**
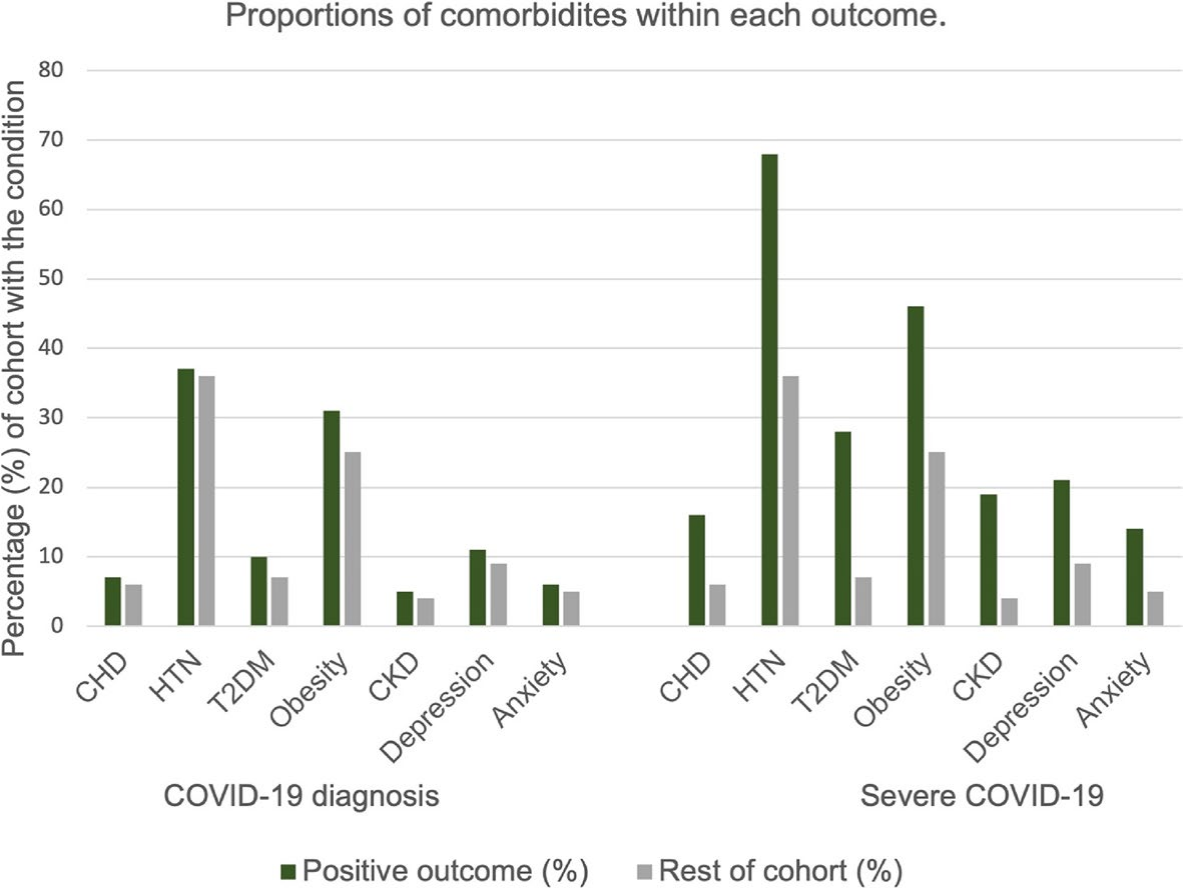
Bar graph representing the proportions of comorbidities within the outcomes COVID-19 diagnosis vs. rest of the cohort and severe COVID-19 vs. rest of the cohort. CHD = Coronary heart disease; HTN = Hypertension; T2DM = Type II diabetes mellitus; CKD = Chronic kidney disease

Those within the cohort with a COVID-19 diagnosis or severe COVID-19 outcome had a higher proportion of each studied comorbidity, compared to the rest of the population (Fig. 2). For example, for T2DM (3013 [10%] diagnoses vs. 29,263 [7%] in the rest of the cohort compared to 891 [28%] severe outcome vs. 313,385 [7%] in the rest of the cohort). Hypertension was the most prevalent comorbidity within both outcomes (11,549 [37%] in the COVID-19 diagnosis cohort and 2153 [68%] in the severe outcome cohort).

### Outcome 1: COVID-19 diagnosis vs. rest of the cohort

An elevated risk of COVID-19 diagnosis was associated with each studied comorbidity. Across all 3 models each comorbidity achieved statistical significance for COVID-19 diagnosis (Table 2; Supplementary Tables 4 and 5, Additional File 1). A diagnosis of CKD is associated with the largest risk increase of COVID-19 diagnosis across all 3 models (Model 1 [OR 1.531, 95% CI 1.453–1.613]; Model 2 [1.717, 1.614–1.826]; Model 3 [1.544, 1.450 – 1.645]). On the other hand, a diagnosis of hypertension had the smallest magnitude of association with COVID-19 diagnosis across all 3 models (Model 1 [1.060, 1.035–1.0862], Model 2 [1.145, 1.112–1.179] and Model 3 [1.080, 1.047–1.113]).

**Table 2:** Logistic regression of COVID-19 diagnosis on comorbidity

	Model 1 (unadjusted) *n* = 472,182	Model 2 (covariates)^a^ *n* = 394974^c^	Model 3 (covariates and comorbidities)^b^ *n* = 396,642
Coronary Heart Disease	1.174 (1.121–1.230)	1.276 (1.209—1.347)	1.153 (1.091 – 1.218)
Hypertension	1.060 (1.035 – 1.086)	1.145 (1.112 – 1.179)	1.080 (1.047 – 1.113)
Type II Diabetes Mellitus	1.521 (1.462 – 1.582)	1.427 (1.360 – 1.497)	1.321 (1.257 – 1.387)
Obesity	1.341 (1.308 – 1.375)	1.258 (1.223 – 1.294)	1.163 (1.129 – 1.198)
Chronic Kidney Disease	1.531 (1.453 – 1.613)	1.717 (1.614 – 1.826)	1.544 (1.450 – 1.645)
Depression	1.309 (1.262 – 1.358)	1.179 (1.131 – 1.229)	1.097 (1.050– 1.147)
Anxiety	1.334 (1.272 – 1.400)	1.272 (1.204 – 1.343)	1.162 (1.097 – 1.231)

Data presented as Odds Ratio (95% Confidence interval)

All *P* values < 0.001

^a^Model 2 was adjusted for ethnicity, sex, age, BMI, Townsend deprivation index, smoking status, qualifications, and pre-tax household income

^b^Model 3 was adjusted for the same covariates as Model 2 (bar BMI) plus the following comorbidities: coronary heart disease, hypertension, type II diabetes mellitus, obesity, chronic kidney disease, depression, and anxiety

^c^n for obesity = 396,642 due to the exclusion of BMI variable

### Outcome 2: severe COVID-19 outcome vs. rest of the cohort

An elevated risk of a severe COVID-19 outcome was associated with each studied comorbidity across all models (Table 3; Supplementary Tables 6 and 7, Additional File 1). A diagnosis of CKD was associated with the largest risk increase in COVID-19 severity across all models (Model 1 [OR 6.427, 95% CI 5.877–7.209]; Model 2 [3.620, 3.238–4.084]; Model 3 [2.454, 2.187–2.752]). In Model 2 and 3, a diagnosis of CHD was associated with the smallest risk increase of a severe COVID-19 outcome (Model 1 [1.687, 1.502–1.894]; Model 2 [1.145, CI: 1.018 – 1.290]) compared with other comorbidities.

**Table 3:** Logistic regression of severe COVID-19 on comorbidity

	Model 1 (unadjusted) *n* = 472,182		Model 2 (covariates)^a^ *n* = 394974^c^		Model 3 (covariates and comorbidities)^b^ *n* = 396,642	
Coronary Heart Disease	3.813 (2.895 – 3.500)	< 0.001	1.687 (1.502 – 1.894)	< 0.001	1.146 (1.018 – 1.290)	0.025
Hypertension	3.738 (3.470 – 4.027)	< 0.001	2.157 (1.966—2.365)	< 0.001	1.666 (1.512 – 1.836)	< 0.001
Type II Diabetes Mellitus	5.423 (5.015 – 5.864)	< 0.001	2.969 (2.685 – 3.283)	< 0.001	2.151 (1.941 – 2.385)	< 0.001
Obesity	2.514 (2.344 – 2.697)	< 0.001	2.080 (1.916 – 2.257)	< 0.001	1.418 (1.298 – 1.548)	< 0.001
Chronic Kidney Disease	6.427 (5.877 – 7.029)	< 0.001	3.620 (3.238 – 4.084)	< 0.001	2.454 (2.187 – 2.752)	< 0.001
Depression	2.737 (2.511 – 2.982)	< 0.001	2.398 (2.163 – 2.658)	< 0.001	1.696 (1.514 – 1.900)	< 0.001
Anxiety	3.223 (2.914 – 3.565)	< 0.001	2.838 (2.515 – 3.202)	< 0.001	1.806 (1.581 – 2.063)	< 0.001

Data presented as Odds Ratio (95% Confidence interval)

^a^Model 2 was adjusted for ethnicity, sex, age, BMI, Townsend deprivation index, smoking status, qualifications, and pre-tax household income

^b^Model 3 was adjusted for the same covariates as model 2 (bar BMI) plus the following comorbidities: coronary heart disease, hypertension, type II diabetes mellitus, obesity, chronic kidney disease, depression, and anxiety

^c^n for obesity = 396,642 due to the exclusion of BMI variable

### Ethnic-specific analysis

**Fig. 3:**
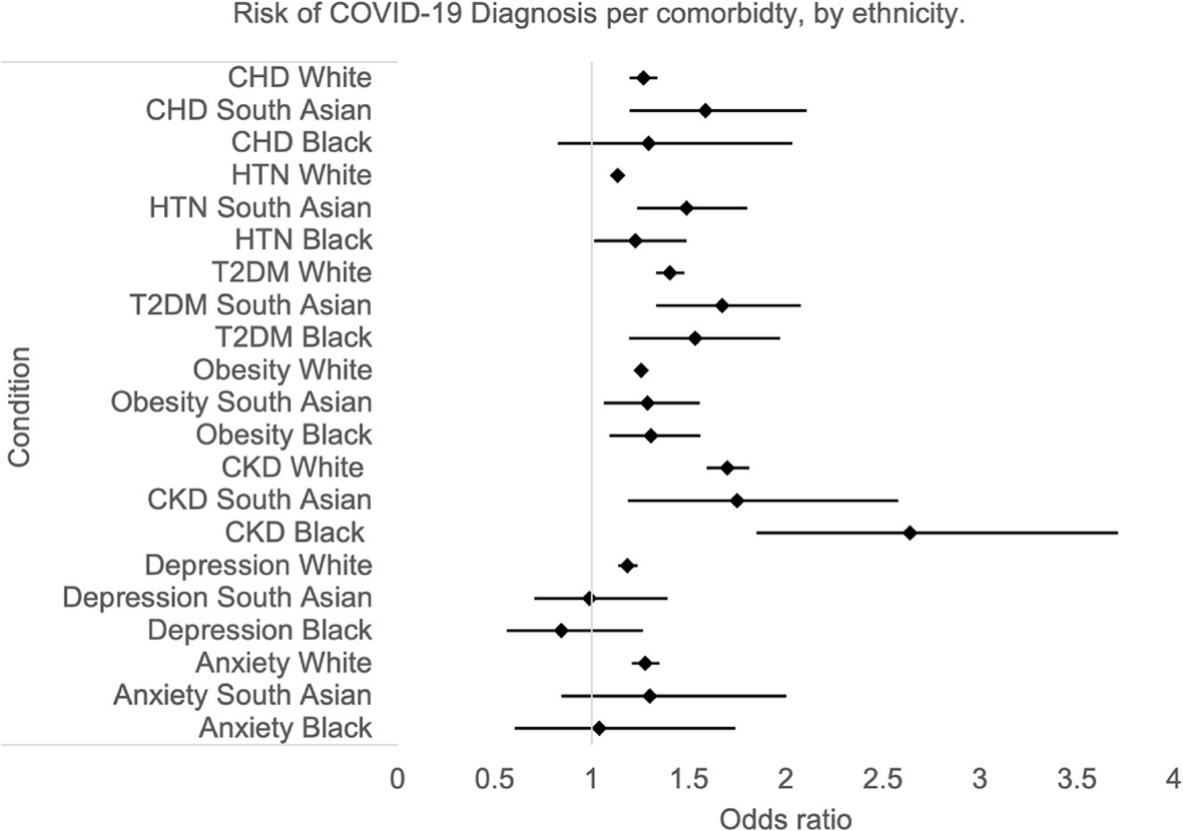
Forest plot showing risk (OR and 95% CI) of COVID-19 diagnosis per comorbidity, by ethnicity. CHD = Coronary heart disease; HTN = Hypertension; T2DM = Type II diabetes mellitus; CKD = Chronic kidney disease

**Fig. 4:**
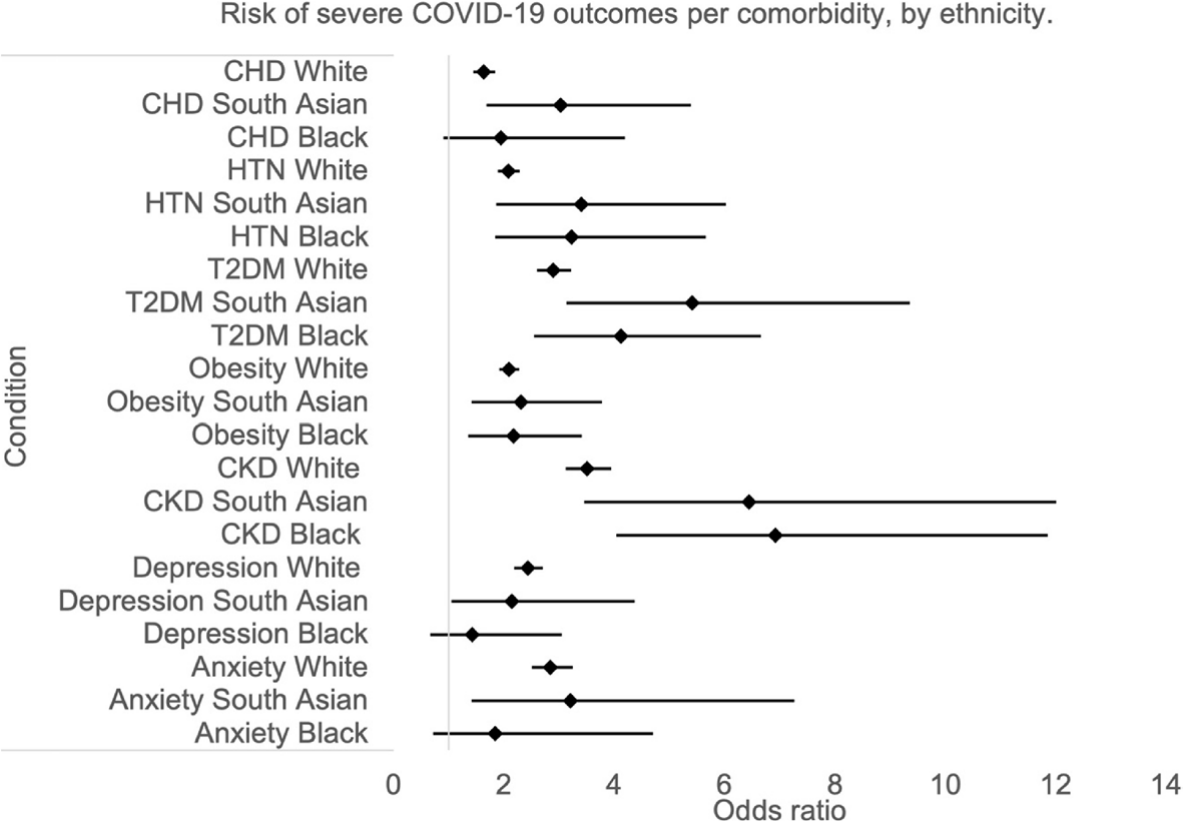
Forest plot showing risk (OR and 95% CI) of severe COVID-19 per comorbidity, by ethnicity. CHD = Coronary heart disease; HTN = Hypertension; T2DM = Type II diabetes mellitus; CKD = Chronic kidney disease

Ethnic-specific analysis showed that when analysing the data by ethnicity (White, South Asian, and Black), an elevated risk of COVID-19 diagnosis and severe COVID-19 outcome was still observed for those with hypertension, T2DM, obesity and CKD. So each ethnic group studied individually showed an elevated risk if they had those comorbidities (Table 4; Figs. 3 and 4). CHD, depression, and anxiety were not observed to have any statistically significant association with COVID-19 diagnosis or severe COVID-19 outcome in black participants (*p* > 0.05). Additionally, a diagnosis of depression or anxiety did not have a statistically significant association with COVID-19 diagnosis for South Asian participants but did have an association with COVID-19 severity (p ≤ 0.05). However, CHD was observed to have an association with COVID-19 diagnosis and severity in South Asian participants (*p* < 0.05).

**Table 4:** Logistic regression of COVID-19 diagnosis and severe COVID-19 on comorbidity, stratified by ethnicity

		White *n* = 376,180		South Asian *n* = 5304		Black *n* = 5703	
		COVID-19 Diagnosis *n* = 24,262	Severe COVID-19 *n* = 2186	COVID-19 Diagnosis *n* = 635	Severe COVID-19 *n* = 67	COVID-19 Diagnosis *n* = 550	Severe COVID-19 *n* = 83
Coronary Heart Disease	OR CI	1.264 1.195–1.336	1.627 1.441–1.837	1.585 1.194–2.105	3.021 1.683–5.390	1.292 0.823–2.030	1.945 0.903–4.191
	*p* value	< 0.001	< 0.001	0.01	< 0.001	1	0.089
Hypertension	OR CI	1.131 1.097–1.166	2.075 1.885–2.284	1.488 1.231–1.799	3.399 1.862–6.206	1.225 1.010–1.487	3.218 1.835–5.645
	*p* value	< 0.001	< 0.001	< 0.001	< 0.001	0.039	< 0.001
Type II Diabetes Mellitus	OR CI	1.402 1.331–1.476	2.890 2.598–3.216	1.671 1.346–2.076	5.412 3.130–9.357	1.531 1.190–1.969	4.119 2.548–6.658
	*p* value	< 0.001	< 0.001	< 0.001	< 0.001	0.001	< 0.001
Obesity	OR CI	1.254 1.218–1.291	2.083 1.913–2.270	1.285 1.061–1.555	2.307 1.409–3.778	1.303 1.090–1.558	2.172 1.348–3.407
	*p* value	< 0.001	< 0.001	< 0.001	0.010	0.004	0.001
Chronic Kidney Disease	OR CI	1.697 1.591–1.809	3.500 3.113–3.936	1.747 1.184–2.579	6.443 3.448–12.001	2.636 1.847–3.707	6.915 4.037–11.846
	*p* value	< 0.001	< 0.001	< 0.001	< 0.001	< 0.001	< 0.001
Depression	OR CI	1.183 1.134–1.235	2.432 2.186–2.705	0.988 0.703–1.388	2.144 1.052–4.370	0.842 0.561–1.263	1.417 0.659–3.049
	*p* value	< 0.001	< 0.001	0.944	0.036	0.405	0.372
Anxiety	OR CI	1.274 1.205–1.347	2.837 2.505–3.241	1.297 0.841–2.000	3.200 1.411–7.259	1.038 0.602–1.739	1.838 0.719–4.703
	*p* value	< 0.001	< 0.001	0.239	0.005	0.886	0.204

Model used was adjusted for ethnicity, sex, age, BMI, Townsend deprivation index, Pre-tax household income, qualifications and smoking status

*OR* Odds Ratio, *CI* 95% Confidence Interval

We saw a statistically significant difference (*p* < 0.05) in the risk of COVID-19 diagnosis and severity between South Asian and White participants with CHD, hypertension and T2DM, with South Asian participants observing a larger risk (Supplementary Table 8, Additional File 1). Additionally, we observed statistically significant difference (*p* < 0.05) in the risk of COVID-19 diagnosis and severity between Black and White participants with hypertension and CKD, with Black participants observing a larger risk.

## Discussion

This study aimed to identify which comorbidities are risk factors for COVID-19 diagnosis and severity (hospitalisation/death due to COVID-19), and to identify whether a difference in this is seen among different ethnic groups. Through a large population-based UK study, our study found that a diagnosis of CHD, hypertension, T2DM, obesity, CKD, depression, and anxiety were independent risk factors for both COVID-19 diagnosis and severe COVID-19. Sub-group analysis showed CHD, hypertension, T2DM, obesity, and CKD to be risk factors for COVID-19 diagnosis and severity in South Asian ethnic groups, with a stronger association seen in the first three comorbidities compared to White ethnic groups. Additionally, it showed that hypertension, CKD, T2DM and obesity were risk factors for COVID-19 diagnosis and severity in Black ethnic groups, with a stronger association seen for the first two comorbidities compared to white ethnic groups. Overall, our results display the increased risk of severe COVID-19 outcomes that certain comorbidities carry and highlight groups of people in need of extra attention such as those with psychiatric conditions and South Asian and Black ethnic groups in the UK.

We observed that a diagnosis of CHD, hypertension, T2DM and obesity were all independent risk factors for COVID-19 diagnosis and severe COVID-19. Obesity, T2DM, hypertension and CHD are interrelated conditions, often co-existing and carrying mutual risk, having similar physiological pathways [21]. Additionally, these conditions are risk factors for CKD [22, 23]. This may account for why we observed CKD having the biggest effect size in our models. However, Model 3 adjusts for all conditions studied and therefore accounts for the influence of related conditions on the effect size of CKD. A similar UKB study by Atkins et al., which exclusively studied older aged individuals (≥ 65), saw similar results to our study [11]. They found a strong association of a diagnosis of T2DM with a severe COVID-19 outcome, but a modest association with hypertension (despite being the most prevalent comorbidity within cohort) [11]. This also matches studies from the USA and China from the start of the pandemic which saw a high prevalence of these conditions in patients with severe COVID-19 outcomes [24]. However, the study by Atkins et al. did not find any association with obesity, CHD and CKD, which our study did [11]. This study only involved older participants and had fewer number of COVID-19 cases, so it might have insufficient statistical power to detect the association. Moreover, as data was from much earlier in the pandemic the testing strategy was restricted to symptomatic hospital inpatients, potentially skewing the results [11]. Although there are not many comparable cohort-studies, research generally supports our findings, with several systematic reviews associating obesity, T2DM, hypertension and CHD with increased risk of infection and severe COVID-19 [5, 6, 25]. A non-UKB cohort study involving 6.9 million people from England associated a higher BMI with severe COVID-19 outcomes [26]. Moreover, conditions such as obesity and T2DM have been linked to an overall increased risk of infection [27, 28]. Literature suggests this increased risk is due to several factors including chronic inflammation, impaired immune response, and enhanced angiotensin-converting enzyme 2 (ACE-2) expression [5, 6, 25, 27, 28]. ACE-2 is the receptor used by SARS-CoV-2 to enter cells [1]. These factors could explain why we observed an increased risk of infection in those with T2DM or those who are obese. This is particularly concerning when considering 28% of the UK population is obese [7]. Moreover, there may be a differential risk in COVID-19 outcomes seen between socio-economics groups with the same comorbidities. For example, in the UK, diabetes was more likely to be mentioned on the COVID-19 death certificate in more deprived areas [7]. Deprivation, which is known to be linked to poorer COVID-19 outcomes, could interact with health conditions to compound this effect.

Our study showed that CKD was associated with COVID-19 diagnosis and severe outcomes. A recent review assessing CKD on COVID-19 outcomes saw an association of CKD with severe COVID-19 but mixed evidence surrounding increased infection [29]. Research also suggests an increased association of CKD and severe pneumonia outcomes [30], and our study found CKD had largest association with COVID-19 diagnosis and severity. Common pathways increasing risk of severe pneumonia could also explain the greater risk of severe COVID-19 outcomes we observed. However, this may be due to the other comorbidities being studied (obesity, T2DM, hypertension and CHD) being risk factors for CKD [22, 23]. Furthermore, Atkins et al. only found a significant association in infection and severity for female CKD patients [11]. This mixed evidence-base highlights the need for further research to elicit the association between CKD and COVID-19. Overall, our findings emphasise the need for targeted policy for groups of the population with these comorbidities, for example prioritisation in vaccination programmes.

UK governmental statistics show South Asian people being the largest minority group in England and Wales in 2019 at 6.1% of the total population (Indian [2.8%], Pakistani [2.26%], Bangladeshi [1.04%]) [19]. This is followed by Black people who make up 3.52% of the total population (African [2.28%], Caribbean [1.02%], Other [0.22%]) [19]. A descriptive review from PHE showed that these minority groups are at increased risk of SARS-CoV-2 infection and severe COVID-19 outcomes when compared to White British people, highlighting the disproportionate effect between groups [4]. The differences were seen after adjusting for demographic and economic factors but did not adjust for occupation or comorbidities, which are known to be influential on COVID-19 outcomes [4]. A previous UKB study by Atkins et al., also found an overall increased risk of severe COVID-19 outcomes for South Asian and Black ethnic groups compared to white ethnic groups [11]. However, our study is one of the first to explore the differential risk between different ethnicities of SARS-CoV-2 and its clinical outcomes, per comorbidity. We observed a stronger association of COVID-19 diagnosis and severe COVID-19 for South-Asian participants with CHD, T2DM and hypertension compared to White people. This is of particular importance, as South Asian people have a much higher prevelance of CHD and T2DM, especially at younger ages than average and in the absence of other factors; this has not only been attributed to socio-economic factors but also genetic and biological factors [4, 8, 31, 32]. Additionally, we saw a stronger association of COVID-19 diagnosis and severe COVID-19 for Black participants with CKD and hypertension, compared to White people. We also found an increased risk for severe COVID-19 outcomes for Black people with T2DM. A non-UKB cohort paper found that Black people with obesity had a higher risk of severe COVID-19 outcomes, compared to white people, which our paper did not [26].This is concerning as Black ethnic groups tend to have higher rates of obesity, hypertension and T2DM [4, 8, 33]. This increased prevalence of comorbidities could partially explain the poorer outcomes experienced by this group of people, alongside adverse socio-economic factors. The descriptive review from PHE stressed the importance of the relationship between comorbidities and ethnicity [4]. Our results highlight the extra attention and targeted care these groups need, as they have an increased prevalence of conditions that put them at higher risk of severe COVID-19 outcomes.

Increased risk of infection in BAME people with these conditions could also be attributed to socioeconomic factors, such as overcrowded households, being more likely to live in economically deprived areas and occupational risk [4, 7]. Occupational risk stems from BAME people more likely to be employed in high-risk environments such as healthcare, hospitality, and retail [7]. BAME people also face barriers in accessing healthcare, for example due to stigma, racism fostering unwelcoming feelings and language barriers [4, 7]. However, there is a nuance to this as not all these groups of people are homogenous, and classification of ethnicities groups a wide range of people together. For example, within the South Asians, Indians suffer a lower risk of CHD than Bangladeshis and Pakistanis and tend to be more socio-economically advantaged [32, 34, 35]. Moreover, Pakistanis and Bangladeshis tend to live in overcrowded, multi-generational households compared to other groups [7, 36]. BAME groups have a lower uptake of the vaccine; particularly Black ethnic groups, which could have modulated outcomes seen [36]. There are many reasons why reduced uptake can be seen, with vaccine safety concerns and mistrust in the development process being a major factor [36, 37]. Other factors include mistrust in health services due to previous discrimination and challenges accessing services (e.g., due to getting to vaccination centres or due to differences in languages) [36, 37]. These various factors contributing to the differential outcomes seen in BAME people emphasise the need for targeted policy to address the unique cultural and societal factors causing the disparities within and between groups. This could involve community action, and boosting positive communication [4, 7, 36]. Policy could also target cash flow for example, self-isolation pay, legal paid time off to get the vaccine or paid accommodation for isolation. Our study’s results highlight the need to enact action, as the differences in outcomes with specific conditions, between ethnicities, is significant. Further work is needed to fully establish the relationship and should assess the effectiveness of future policy targeted at these groups.

In the England, it is estimated that 15.7% (1 in 6) of adults over 16 have symptoms of depression and anxiety [10]. Our study found an association of a diagnosis of depression and anxiety with SARS-CoV-2 infection and severe COVID-19, which is worrying due to the prevalence of these conditions. A UKB study by Yang et al., found that a diagnosis of psychiatric disorders pre-pandemic was associated with increased risk of COVID-19 (infection and severe outcomes) [12]. The results were similar to our study when depression and anxiety were analysed alone [12]. Atkins et al. also found a similar association between depression and severe COVID-19 in their UKB analysis [11]. Our study had a larger sample size regarding COVID-19 cases than the previous UKB studies and therefore can more concretely confirm the findings from these previous analyses. Although there are not many similar studies, the link between psychiatric disorders and infections has been studied showing associations [9]. The reasons for this are not fully understood but have been previously attributed to biological mechanisms which compromise immunity, as well as poor health behaviours and socioeconomic factors [12]. More research needs to be undertaken to fully establish the increased risk of COVID-19 linked to mental health conditions, but the current evidence suggests an association. Our study did not find any differences in risk between people with anxiety and depression in different ethnicities. Yang et al., also found no differential risk between different ethnic groups [12]. Although both studies found no significant differences, the dataset had relatively small sample sizes for these conditions and therefore more research needs to be done to ascertain the true relationship. Overall, our results highlight the need to assess the role of mental health when managing and creating policy around COVID-19.

This is one of the first studies to analyse the differential risk of COVID-19 outcomes that different comorbidities confer, by ethnicity. A major strength of this study is the use of original UKB dataset; the large sample size and breadth of data allowed for a comprehensive analysis to be undertaken. It allowed for a range of confounders to be accounted for, that are known to influence COVID-19 outcomes, such as age, sex, deprivation, and smoking status [3, 4]. This increased the internal validity of the results, allowing for more accurate and reliable conclusions to be drawn. Moreover, information bias was also reduced through the data collection method, and nature of the longitudinal study.

This study had several limitations. Firstly, as this is a UK-based study the results are not globally generalisable. Furthermore, the sample is not representative of the UK population, questioning the external validity of the study of the whole-group analyses [38]. Whilst proportions of White and Black ethnic groups were comparable with the 2001 UK census, South Asians were underrepresented in the original sample (1.6% of total UKB cohort vs 2.5% of 2001 population) [38]. Compounding this as populations are dynamic overtime, South Asians become severely underrepresented (6.1% of 2019 population estimate vs. or 2–3% in our analyses) [19, 38]. This reduces the reliability of conclusions that the whole-group analyses can be for specific groups – they will mainly be applicable to White ethnic groups. The sub-group analysis accounts for this, but due to underrepresentation the samples are small for the minority ethnic groups relative to the White ethnic group, as can be seen by the large confidence intervals in this analysis. This reduces the reliability of the conclusions drawn. On the other hand, Black ethnic groups are better represented in the whole-group analyses (3.5% of the population vs. 2–3% in the analysis). Small sample sizes meant it was not possible to differentiate within ethnic groups too (e.g., differentiating risk between Pakistani and Indian participants within the cohort). Other misrepresentation includes the cohort being older than the general population (as UKB recruited people > 40 years of age exclusively) and healthier than the general population [38]. Secondly, in our analysis we did not account for factors that would affect COVID-19 outcomes over the studied period (January 2020 to March 2022), such as vaccine rollout programmes, COVID-19 treatments, and different variants of the virus. Future analyses could adjust for these to increase internal validity and reliability. For instance, adjusting using data on the predominant strains at different time could be increase the reliability as different strains of the virus caused different outcomes. As different strains likely caused differential outcomes, there would have been an impact on COVID-19 diagnosis and severity. Thirdly, the data on certain covariates and comorbidities could be utilised in more accurate ways. For example, for Obesity levels were calculated from combining baseline BMI data with hospital records; however, this may misrepresent the amount of obese people in the data. Moreover, we used Townsend deprivation index for a proxy of household overcrowding (amongst other factors), but UKB have data on the number of people in households which would have allowed for a more accurate analysis. This would be particularly important in the sub-group analysis as we know certain ethnic groups have higher risk due to overcrowded multigenerational households, which could have been accounted for. If further analyses were done, factors such as job type could be included, as certain ethnic groups are more likely to have certain occupations that put them at higher risk of SARS-CoV-2 infection [4, 7]. Finally, further data on certain conditions known to affect COVID-19 outcomes could have been added into our study. For example, respiratory illnesses are known to be linked with worse COVID-19 outcomes [4, 5]. This means our results from Model 3, which was adjusted for multiple comorbidities, may have been obscured and not as reliable.

## Conclusion

In conclusion, in the UKB cohort, certain comorbidities are associated with increased risk of SARS-CoV-2 infection and consequential hospitalisation and/or death. This includes CHD, hypertension, T2DM, obesity, CKD, depression, and anxiety. Further research is needed to elicit the effect of CKD, depression, and anxiety specifically. However, inclusion of these groups when creating future public health policy and treatment regarding COVID-19 is essential, especially for psychiatric disorders. We also observed a stronger association between South Asian people with CHD, hypertension and T2DM, and Black people with CKD and hypertension with COVID-19 diagnosis and severe COVID-19 compared to White people. Although further research is needed, the difference observed between ethnic groups supports the need for targeted policies and intervention, accounting for the unique reasons that these groups have higher risks; especially as these groups often suffer from higher rates of these comorbidities.

### Supplementary Information

**Additional file 1: Supplementary Table 1.** Prevalence of studied comorbidities stratified by ethnicity, presented as N (N%). **Supplementary Table 2.** Collinearity between all studied covariates, showing the Variance Inflation Factor. **Supplementary Table 3.** Collinearity test between each variable, showing the Pearson correlation measure. **Supplementary Table 4.** Logistic regression of COVID-19 diagnosis on comorbidity for Model 2, data presented as OR (95%CI). **Supplementary Table 5.** Logistic regression of risk of COVID-19 diagnosis for model 3, presented as OR (95% CI). **Supplementary Table 6.** Logistic regression of severe COVID-19 on comorbidity for Model 2. **Supplementary Table 7.** Logistic regression of risk of severe COVID-19 for model 3, presented as OR (95% CI). **Supplementary Table 8.** Table displaying p values for the difference between odds ratios for risk of infection and severity per comorbidity, stratified by ethnicity.

## Data Availability

The data that support the findings of this study are available from UK Biobank to any bona fide researchers who can apply to access the UK Biobank research resource. Researchers can apply via this link https://www.ukbiobank.ac.uk/enable-your-research/apply-for-access. However, requests can be forwarded to the corresponding authors regarding the particular datasets used and results produced in this study under the Material Transfer Agreement with UK Biobank.

## Abbreviations

SARS-CoV-2: Severe Acute Respiratory Syndrome Coronavirus 2
COVID-19: Coronavirus Disease 2019
T2DM: Type II Diabetes Mellitus
CKD: Chronic Kidney Disease
BAME: Black, Asian, and Minority Ethnic
CHD: Coronary Heart Disease
UKB: UK Biobank
RT-PCR: Reverse-Transcriptase Polymerase Chain Reaction
PHE: Public Health England
SAIL: Secure Anonymised Information Linkage
ICD-10: International Classification of Diseases 10^th^ revision
BMI: Body Mass Index
OR: Odds Ratio
95% CI: 95% Confidence Interval
HTN: Hypertension
ACE-2: Angiotensin-Converting Enzyme 2
