# Supplementary material for: Comorbidities associated with the severity of COVID-19, and differences across ethnic groups: a UK Biobank cohort study
Source: BMC Public Health. 2023 Aug 17;23:1566. doi: 10.1186/s12889-023-16499-6 (PMC10436456; doi:10.1186/s12889-023-16499-6)
Supplement: Supplementary file 1 — Additional file 1: Supplementary Table 1. Prevalence of studied comorbidities stratified by ethnicity, presented as N (N%). Supplementary Table 2. Collinearity between all studied covariates, showing the Variance Inflation Factor. Supplementary Table 3. Collinearity test between each variable, showing the Pearson correlation measure. Supplementary Table 4. Logistic regression of COVID-19 diagnosis on comorbidity for Model 2, data presented as OR (95%CI). Supplementary Table 5. Logistic regression of risk of COVID-19 diagnosis for model 3, presented as OR (95% CI). Supplementary Table 6. Logistic regression of severe COVID-19 on comorbidity for Model 2. Supplementary Table 7. Logistic regression of risk of severe COVID-19 for model 3, presented as OR (95% CI). Supplementary Table 8. Table displaying p values for the difference between odds ratios for risk of infection and severity per comorbidity, stratified by ethnicity. [file 12889_2023_16499_MOESM1_ESM.pdf]

Additional File 1

Supplementary Table 1: Prevalence of studied comorbidities stratified by ethnicity, presented as N (N%).

	White		South Asian		Black	
	Has comorbidity	Does not have Comorbidity	Has comorbidity	Does not have Comorbidity	Has comorbidity	Does not have Comorbidity
Coronary Heart Disease	25825 (6%)	417898 (94%)	726 (9%)	6933 (91%)	316 (4%)	7429 (96%)
Hypertension	158904 (36%)	284819 (64%)	3312 (43%)	4347 (57%)	3614 (47%)	4130 (53%)
Type II Diabetes Mellitus	28001 (6%)	415722 (94%)	1612 (21%)	6047 (79%)	1144 (15%)	6600 (85%)
Obesity	111229 (25%)	332494 (75%)	1801 (24%)	5858 (76%)	3165 (41%)	4579 (59%)
Chronic Kidney Disease	16152 (4%)	427571 (96%)	366 (5%)	7293 (95%)	389 (5%)	7355 (95%)
Depression	40000 (9%)	403723 (91%)	540 (7%)	7119 (93%)	466 (6%)	7278 (94%)
Anxiety	22033 (5%)	421690 (95%)	260 (3%)	7399 (97%)	249 (4%)	7495 (96%)

Supplementary Table 2: Collinearity between all studied covariates, showing the Variance Inflation Factor.

	Variance Inflation Factor
Coronary Heart Disease	1.088
Hypertension	1.275
Type II Diabetes Mellitus	1.144
Obesity	2.408
Chronic Kidney Disease	1.070
Depression	1.138
Anxiety	1.117
Age	1.291
Body Mass Index	2.488
Townsend Deprivation Index	1.141
Sex	1.061
Ethnicity	1.048
Smoking Status	1.049
Qualifications	1.235
Pre-tax Household Income	1.386

Supplementary Table 3: Collinearity test between each variable, showing the Pearson correlation measure.

	TDI	Pre-tax Household Income	Qualifications	Sex	Ethnicity	Age	BMI	CHD	HTN	T2DM	Obesity	CKD	Depression	Anxiety
TDI	1	-0.231 p<0.001	0.115 p<0.001	0.008 p<0.001	0.188 p<0.001	-0.104 p<0.001	0.092 p<0.001	0.039 p<0.001	0.054 p<0.001	0.083 p<0.001	0.091 p<0.001	0.028 p<0.001	0.076 p<0.001	0.043 p<0.001
Pre-tax household income	-0.231 p<0.001	1	-0.394 p<0.001	0.078 p<0.001	-0.048 p<0.001	-0.315 p<0.001	-0.101 p<0.001	-0.100 p<0.001	-0.174 p<0.001	-0.117 p<0.001	-0.094 p<0.001	-0.094 p<0.001	-0.113 p<0.001	-0.084 p<0.001
Qualifications	0.115 p<0.001	-0.394 p<0.001	1	-0.002 P=0.215	-0.023 p<0.001	0.257 p<0.001	0.138 p<0.001	0.138 p<0.001	0.167 p<0.001	0.105 p<0.001	0.114 p<0.001	0.089 p<0.001	0.044 p<0.001	0.049 p<0.001
Sex	0.008 p<0.001	0.078 p<0.001	-0.002 p<0.215	1	-0.005 p<0.001	0.012 p<0.001	0.077 p<0.001	0.122 p<0.001	0.095 p<0.001	0.070 p<0.001	0.019 p<0.001	0.018 p<0.001	-0.065 p<0.001	-0.052 p<0.001
Ethnicity	0.188 p<0.001	-0.048 p<0.001	-0.023 p<0.001	-0.005 p<0.001	1	-0.120 p<0.001	0.015 p<0.001	-0.004 p=0.014	0.015 p<0.001	0.061 p<0.001	0.014 p<0.001	0.002 p=0.238	-0.010 p<0.001	-0.014 p<0.001
Age	-0.104 p<0.001	-0.315 p<0.001	0.257 p<0.001	0.012 p<0.001	-0.120 p<0.001	1	0.043 p<0.001	0.143 p<0.001	0.288 p<0.001	0.109 p<0.001	0.024 p<0.001	0.138 p<0.001	-0.025 p<0.001	0.010 p<0.001
BMI	0.092	-0.101	0.138 p<0.001	0.077	0.015	0.043	1	0.091	0.274	0.240	0.763	0.093	0.093	0.035

	$p<0.001$	$p<0.001$		$p<0.001$	$p<0.001$	$p<0.001$		$p<0.001$	$p<0.001$	$p<0.001$	$p<0.001$	$p<0.001$	$p<0.001$	$p<0.001$
CHD	0.039 $p<0.001$	-0.100 $p<0.001$	0.138 $p<0.001$	0.122 $p<0.001$	-0.004 $p=0.014$	0.143 $p<0.001$	0.091 $p<0.001$	1	0.214 $p<0.001$	0.151 $p<0.001$	0.082 $p<0.001$	0.124 $p<0.001$	0.038 $p<0.001$	0.040 $p<0.001$
HTN	0.054 $p<0.001$	-0.174 $p<0.001$	0.167 $p<0.001$	0.095 $p<0.001$	0.015 $p<0.001$	0.288 $p<0.001$	0.274 $p<0.001$	0.214 $p<0.001$	1	0.254 $p<0.001$	0.234 $p<0.001$	0.184 $p<0.001$	0.075 $p<0.001$	0.083 $p<0.001$
T2DM	0.083 $p<0.001$	-0.117 $p<0.001$	0.105 $p<0.001$	0.070 $p<0.001$	0.061 $p<0.001$	0.109 $p<0.001$	0.240 $p<0.001$	0.151 $p<0.001$	0.254 $p<0.001$	1	0.212 $p<0.001$	0.177 $p<0.001$	0.077 $p<0.001$	0.058 $p<0.001$
Obesity	0.091 $p<0.001$	-0.094 $p<0.001$	0.114 $p<0.001$	0.019 $p<0.001$	0.014 $p<0.001$	0.024 $p<0.001$	0.763 $p<0.001$	0.082 $p<0.001$	0.234 $p<0.001$	0.212 $p<0.001$	1	0.089 $p<0.001$	0.079 $p<0.001$	0.043 $p<0.001$
CKD	0.028 $p<0.001$	-0.094 $p<0.001$	0.089 $p<0.001$	0.018 $p<0.001$	0.002 $p=0.238$	0.138 $p<0.001$	0.093 $p<0.001$	0.124 $p<0.001$	0.184 $p<0.001$	0.177 $p<0.001$	0.089 $p<0.001$	1	0.056 $p<0.001$	0.059 $p<0.001$
Depression	0.076 $p<0.001$	-0.113 $p<0.001$	0.044 $p<0.001$	-0.065 $p<0.001$	-0.010 $p<0.001$	-0.025 $p<0.001$	0.093 $p<0.001$	0.038 $p<0.001$	0.075 $p<0.001$	0.077 $p<0.001$	0.079 $p<0.001$	0.056 $p<0.001$	1	0.316 $p<0.001$
Anxiety	0.043 $p<0.001$	-0.084 $p<0.001$	0.049 $p<0.001$	-0.052 $p<0.001$	-0.014 $p<0.001$	0.010 $p<0.001$	0.035 $p<0.001$	0.040 $p<0.001$	0.083 $p<0.001$	0.058 $p<0.001$	0.043 $p<0.001$	0.059 $p<0.001$	0.316 $p<0.001$	1

TDI=Townsend Deprivation Index; CHD=Coronary heart disease; HTN=Hypertension; T2DM=Type II diabetes mellitus; CKD=Chronic kidney disease.

Supplementary Table 4: Logistic regression of COVID-19 diagnosis on comorbidity for Model 2, data presented as OR (95%CI).

	CHD n = 394974	HTN n = 394974	T2DM n = 394974	Obesity n = 396642	CKD n = 394974	Depression n = 394974	Anxiety n = 394974
Comorbidity	1.276 (1.209 – 1.347) *p*<0.001	1.145 (1.112 – 1.179) *p*<0.001	1.427 (1.360 – 1.497) *p*<0.001	1.258 (1.223 – 1.294) *p*<0.001	1.717 (1.614 – 1.826) *p*<0.001	1.179 (1.131 – 1.229) *p*<0.001	1.272 (1.204 – 1.343) *p*<0.001
Age	0.951 (0.949 – 0.953) *p*<0.001	0.950 (0.948 – 0.952) *p*<0.001	0.951 (0.949 – 0.953) *p*<0.001	0.952 (0.951 – 0.954) *p*<0.001	0.950 (0.949 – 0.952) *p*<0.001	0.952 (0.951 – 0.954) *p*<0.001	0.952 (0.950 – 0.954) *p*<0.001
Townsend Deprivation Index	1.019 (1.014 – 1.023) *p*<0.001	1.019 (1.014 – 1.023) *p*<0.001	1.018 (1.014 – 1.023) *p*<0.001	1.020 (1.016 – 1.024) *p*<0.001	1.019 (1.014 – 1.023) *p*<0.001	1.019 (1.014 – 1.023) *p*<0.001	1.019 (1.015 – 1.023) *p*<0.001
Body Mass Index	1.025 (1.023 – 1.028) *p*<0.001	1.023 (1.020 – 1.025) *p*<0.001	1.021 (1.019 – 1.024) *p*<0.001	N/A*	1.024 (1.022 – 1.027) *p*<0.001	1.025 (1.023 – 1.028) *p*<0.001	1.026 (1.023 – 1.028) *p*<0.001
Sex – Female	1.00	1.00	1.00	1.00	1.00	1.00	1.00
Sex – Male	1.079 (1.052 – 1.108) *p*<0.001	1.082 (1.055 – 1.111) *p*<0.001	1.083 (1.055 – 1.111) *p*<0.001	1.108 (1.080 – 1.136) *p*<0.001	1.091 (1.063 – 1.119) *p*<0.001	1.102 (1.074 – 1.130) *p*<0.001	1.100 (1.072 – 1.128) *p*<0.001
Ethnicity – White	1.00	1.00	1.00	1.00	1.00	1.00	1.00
Ethnicity – South Asian	1.667 (1.530 – 1.816) *p*<0.001	1.662 (1.525 – 1.811) *p*<0.001	1.604 (1.472 – 1.748) *p*<0.001	1.685 (1.548 – 1.833) *p*<0.001	1.670 (1.533 – 1.819) *p*<0.001	1.696 (1.557 – 1.848) *p*<0.001	1.693 (1.554 – 1.854) *p*<0.001
Ethnicity – Black	1.103 (1.006 – 1.209) *p* 0.036	1.084 (0.989- 1.188) *p* 0.084	1.081 (0.986 – 1.185) *p* 0.095	1.128 (1.031 – 1.235) *p* 0.009	1.091 (0.995 – 1.195) *p* 0.063	1.116 (1.018 – 1.223) *p* 0.019	1.110 (1.013 – 1.216) *p* 0.026
Ethnicity – Other	1.026 (0.942 – 1.117) *p* 0.559	1.020 (0.937 – 1.111) *p* 0.649	1.008 (0.925 – 1.098) *p* 0.862	1.025 (0.942 – 1.116) *p* 0.564	1.024 (0.940 – 1.115) *p* 0.592	1.033 (0.949 – 1.125) p <0.454	1.033 (0.948 – 1.125) *p* 0.463

Smoking Status – Never	1.00	1.00	1.00	1.00	1.00	1.00	1.00
Smoking Status – Previous	1.131 (1.100 – 1.164) *p*<0.001	1.133 (1.102 - 1.165) *p*<0.001	1.132 (1.100 – 1.164) *p*<0.001	1.141 (1.110 -1.173) *p*<0.001	1.134 (1.103 – 1.166) *p*<0.001	1.133 (1.102 – 1.166) *p*<0.001	1.135 (1.104 – 1.167) *p*<0.001
Smoking Status - Current	0.960 (0.919 – 1.002) *p* 0.063	0.963 (0.922 – 1.005) *p* 0.087	0.960 (0.919 – 1.098) *p* 0.061	0.957 (0.917 – 0.999) *p* 0.046	0.965 (0.924 (1.007) *p* 0.102	0.956 (0.916 – 0.999) *p* 0.043	0.960 (0.919 – 1.002) *p* 0.064
Qualifications – Degree	1.00	1.00	1.00	1.00	1.00	1.00	1.00
Qualifications – School	1.323 (1.282 – 1.366) *p*<0.001	1.321 (1.280 – 1.363) *p*<0.001	1.323 (1.282 – 1.366) *p*<0.001	1.335 (1.294 – 1.378) *p*<0.001	1.323 (1.281 – 1.365) *p*<0.001	1.327 (1.286 – 1.369) *p*<0.001	1.325 (1.284 – 1.367) p <0.001
Qualifications – Professional	1.447 (1.384 – 1.513) *p*<0.001	1.445 (1.382 – 1.510) *p*<0.001	1.446 (1.383 – 1.511) *p*<0.001	1.464 (1.400 – 1.530) *p*<0.001	1.446 (1.383 – 1.511) *p*<0.001	1.452 (1.389 – 1.518) *p*<0.001	1.450 (1.387 – 1.515) *p*<0.001
Qualifications – None	1.771 (1.673 – 1.788) *p*<0.001	1.709 (1.635 – 1.786) *p*<0.001	1.710 (1.636 – 1.787) *p*<0.001	1.741 (1.666 – 1.819) *p*<0.001	1.707 (1.633 – 1.785) *p*<0.001	1.726 (1.651 – 1.804) *p*<0.001	1.720 (1.646 – 1.798) *p*<0.001
Pre-tax Household Income - <£18,000	1.063 (0.955 – 1.136) *p 0.069*	1.061 (0.993 – 1.134) *p* 0.080	1.055 (0.987 1.127) *p* 0.114	1.078 (1.009 – 1.152) *p* 0.025	1.058 (0.990 – 1.130) *p* 0.098	1.051 (0.981 – 1.114) *p* 0.145	1.056 (0.988 – 1.129) *p* 0.108
Pre-tax Household Income - £18,000 to £30,999	1.054 (0.989 – 1.123) *p* 0.106	1.050 (0.986 – 1.119) *p* 0.129	1.050 (0. 987 – 1.127) *p* 0.133	1.062 (0.997 – 1.131) *p* 0.064	1.052 (0.987 – 1.121) *p* 0.116	1.046 (0.981 – 1.114) *p* 0.168	1.048 (0.983 – 1.116) *p* 0.148
Pre-tax Household Income - £31,000 to £51,999	1.093 (1.029 – 1.163) *p* 0.004	1.091 (1.026 – 1.159) *p* 0.006	1.092 (1.027 – 1.161) *p* 0.005	1.101 (1.036 – 1.170) *p* 0.002	1.092 (1.028 – 1.161) *p* 0.005	1.088 (1.023 – 1.157) *p* 0.007	1.089 (1.024 – 1.158) *p* 0.006
Pre-tax Household Income - £52,000 to £100,000	1.041 (0.978 – 1.107) *p* 0.205	1.040 (0.977 – 1.106) *p* 0.218	1.040 (0.987 – 1.106) *p* 0.213	1.044 (0.981 – 1.110) *p* 0.173	1.040 (0.977 – 1.106) *p* 0.217	1.039 (0.976 – 1.105) *p* 0.230	1.038 (0.976 – 1.105) *p* 0.232

Pre-tax Household Income - >£100,000	1.00	1.00	1.00	1.00	1.00	1.00	1.00

CHD=Coronary heart disease; HTN=Hypertension; T2DM=Type II diabetes mellitus; CKD=Chronic kidney disease.

*BMI not included in this analysis due to collinearity with obesity.

Supplementary Table 5: Logistic regression of risk of COVID-19 diagnosis for model 3, presented as OR (95% CI)

	Odds Ratio (95% Confidence Interval)	*p* value
Coronary Heart Disease	1.153 (1.091 – 1.218)	<0.001
Hypertension	1.080 (1.047 – 1.113)	<0.001
Type II Diabetes Mellitus	1.321 (1.257 – 1.387)	<0.001
Obesity	1.163 (1.129 – 1.198)	<0.001
Chronic Kidney Disease	1.544 (1.450 – 1.645)	<0.001
Depression	1.097 (1.050– 1.147)	<0.001
Anxiety	1.162 (1.097 – 1.231)	<0.001
Age	0.949 (0.947 – 0.950)	<0.001
Townsend Deprivation Index	1.018 (1.013 – 1.022)	<0.001
Sex – Female	1.00	-
Sex - Male	1.087 (1.060 – 1.116)	<0.001
Ethnicity – White	1.00	-
Ethnicity – South Asian	1.600 (1.470 – 1.742)	<0.001
Ethnicity – Black	1.112 (1.016 – 1.218)	0.021
Ethnicity – Other	1.013 (0.931 – 1.103	0.759
Smoking Status – Never	1.00	-
Smoking Status – Previous	1.127 (1.096 – 1.159)	<0.001
Smoking Status - Current	0.938 (0.898 – 0.979)	0.003
Qualifications – Degree	1.00	-
Qualifications – School	1.326 (1.286 – 1.368)	<0.001
Qualifications – Professional	1.446 (1.383 – 1.511)	<0.001
Qualifications – None	1.698 (1.625 – 1.775)	<0.001
Pre-tax Household Income - <£18000	1.019 (0.954 – 1.089)	0.575
Pre-tax Household Income - £18000 to £30999	1.038 (0.974 – 1.105)	0.254
Pre-tax Household Income - £31000 to £51999	1.086 (1.022 – 1.155)	0.008
Pre-tax Household Income - £52000 to £100000	1.037 (0.975 – 1.103)	0.253
Pre-tax Household Income - >£100000	1.00	-

n = 396642.

Supplementary Table 6: Logistic regression of severe COVID-19 on comorbidity for Model 2

	CHD<br>n = 394974	HTN<br>n = 394974	T2DM<br>n = 394974	Obesity<br>n = 396642	CKD<br>n = 394974	Depression<br>n = 394974	Anxiety<br>n = 394974
Comorbidity	1.687 (1.502 – 1.894) *p*<0.001	2.157 (1.966 – 2.365) *p*<0.001	2.969 (2.685 – 3.283) *p*<0.001	2.080 (1.916 – 2.257) *p*<0.001	3.620 (3.238 – 4.048) *p*<0.001	2.398 (2.163 – 2.658) *p*<0.001	2.838 (2.515 – 3.202) *p*<0.001
Age	1.041 (1.035 – 1.048) *p*<0.001	1.031 (1.025 – 1.038) *p*<0.001	1.037 (1.031 – 1.044) *p*<0.001	1.044 (1.037 – 1.050) *p*<0.001	1.035 (1.028 – 1.041) *p*<0.001	1.048 (1.042 – 1.055) *p*<0.001	1.046 (1.040 – 1.053) *p*<0.001
Townsend Deprivation Index	1.063 (1.049 – 1.077) *p*<0.001	1.061 (1.047 – 1.075) *p*<0.001	1.058 (1.045 – 1.072) *p*<0.001	1.066 (1.053 – 1.080) *p*<0.001	1.061 (1.047 – 1.075) *p*<0.001	1.061 (1.047 – 1.075) *p*<0.001	1.063 (1.049 – 1.077) *p*<0.001
Body Mass Index	1.072 (1.065 – 1.080) *p*<0.001	1.057 (1.049 – 1.065) *p*<0.001	1.051 (1.043 – 1.059) *p*<0.001	N/A*	1.066 (1.058 – 1.074) *p*<0.001	1.070 (1.062 – 1.077) *p*<0.001	1.073 (1.065 – 1.081) *p*<0.001
Sex – Female	1.00	1.00	1.00	1.00	1.00	1.00	1.00
Sex – Male	1.701 (1.562 – 1.852) *p*<0.001	1.665 (1.529 – 1.812) *p*<0.001	1.655 (1.520 – 1.801) *p*<0.001	1.766 (1.624 – 1.920) *p*<0.001	1.754 (1.612 – 1.909) *p*<0.001	1.874 (1.722 – 2.040) *p*<0.001	1.861 (1.710 – 2.026) *p*<0.001
Ethnicity – White	1.00	1.00	1.00	1.00	1.00	1.00	1.00
Ethnicity – South Asian	2.362 (1.840 – 3.031) *p*<0.001	2.226 (1.735 – 2.858) *p*<0.001	1.873 (1.456 – 2.409) *p*<0.001	2.427 (1.901 – 3.098) *p*<0.001	2.313 (1.801 – 2.970) *p*<0.001	2.589 (2.017 – 3.323) *p*<0.001	2.542 (1.980 – 2.954) *p*<0.001
Ethnicity – Black	2.231 (1.771 – 2.809) *p*<0.001	2.016 (1.601 – 2.540) *p*<0.001	1.975 (1.567 – 2.489) *p*<0.001	2.236 (1.780 – 2.808) *p*<0.001	2.096 (1.663 – 2.642) *p*<0.001	2.438 (1.935 – 3.072) *p*<0.001	2.345 (1.861 – 2.954) *p*<0.001
Ethnicity – Other	1.367 (1.041 – 1.796) *p* 0.025	1.312 (0.999 – 1.724) *p* 0.051	1.206 (0.917 – 1.585) *p* 0.180	1.360 (1.040 – 1.778) *p* 0.025	1.352 (1.028 – 1.777) *p* 0.031	1.429 (1.088 – 1.878) *p* 0.010	1.420 (1.081 – 1.865) *p* 0.012

Smoking Status – Never	1.00	1.00	1.00	1.00	1.00	1.00	1.00
Smoking Status – Previous	1.262 (1.153 – 1.381) *p*<0.001	1.257 (1.148 – 1.357) *p*<0.001	1.247 (1.139 – 1.365) *p*<0.001	1.296 (1.185 – 1.418) *p*<0.001	1.269 (1.159 – 1.389) *p*<0.001	1.261 (1.152 – 1.380) *p*<0.001	1.277 (1.167 – 1.397) *p*<0.001
Smoking Status - Current	1.677 (1.483 – 1.897) *p*<0.001	1.684 (1.488 – 1.905) *p*<0.001	1.639 (1.448 – 1.854) *p*<0.001	1.669 (1.477 – 1.885) *p*<0.001	1.688 (1.492 – 1.910) *p*<0.001	1.608 (1.421 – 1.820) *p*<0.001	1.657 (1.465 – 1.874) *p*<0.001
Qualifications – Degree	1.00	1.00	1.00	1.00	1.00	1.00	1.00
Qualifications – School	1.228 (1.095 – 1.377) *p*<0.001	1.207 (1.077 – 1.354) *p* 0.001	1.220 (1.088 – 1.368) *p*<0.001	1.247 (1.114 – 1.398) *p*<0.001	1.220 (1.088 – 1.368) *p*<0.001	1.252 (1.117 – 1.404) *p*<0.001	1.240 (1.106 – 1.391) *p*<0.001
Qualifications – Professional	1.356 (1.178 – 1.560) *p*<0.001	1.332 (1.157 – 1.533) *p*<0.001	1.339 (1.164 – 1.542) *p*<0.001	1.394 (1.212 – 1.602) *p*<0.001	1.342 (1.166 – 1.545) *p*<0.001	1.390 (1.208 – 1.599) *p*<0.001	1.372 (1.192 – 1.578) *p*<0.001
Qualifications – None	1.636 (1.440 – 1.859) *p*<0.001	1.598 (1.407 – 1.815) *p*<0.001	1.612 (1.419 – 1.832) *p*<0.001	1.676 (1.476 – 1.902) *p*<0.001	1.607 (1.414 – 1.827) *p*<0.001	1.696 (1.493 – 1.927) *p*<0.001	1.667 (1.468 – 1.894) *p*<0.001
Pre-tax Household Income - <£18,000	1.993 (1.502 – 2.643) *p*<0.001	1.922 (1.449 – 2.549) *p*<0.001	1.897 (1.430 – 2.517) *p*<0.001	2.132 (1.608 – 2.825) *p*<0.001	1.949 (1.469 – 2.586) *p*<0.001	1.798 (1.355 – 2.386) *p*<0.001	1.880 (1.417 – 2.494) *p*<0.001
Pre-tax Household Income - £18,000 to £30,999	1.393 (1.052 – 1.845) *p* 0.021	1.351 (1.020 – 1.789) *p* 0.036	1.352 (1.021 – 1.791) *p* 0.035	1.439 (1.087 – 1.906) *p* 0.011	1.387 (1.047 – 1.838) *p* 0.023	1.311 (0.990 – 1.736) *p* 0.059	1.339 (1.011 – 1.774) *p* 0.042
Pre-tax Household Income - £31,000 to £51,999	1.318 (0.996 – 1.745) *p* 0.054	1.283 (0.969 – 1.700) *p* 0.082	1.294 (0.977 – 1.713) *p* 0.072	1.347 (1.018 – 1.783) *p* 0.037	1.315 (0.993 – 1.741) *p* 0.056	1.266 (0.956 – 1.676) *p* 0.100	1.283 (0.969 – 1.699) *p* 0.082
Pre-tax Household Income - £52,000 to £100,000	1.180 (0.884 – 1.575) *p* 0.262	1.165 (0.872 – 1.555) *p* 0.301	1.167 (0.874 – 1.557) *p* 0.296	1.196 (0.896 – 1.596) *p* 0.225	1.171 (0.877 – 1.563) *p* 0.285	1.157 (0.866 – 1.544) *p* 0.323	1.163 (0.871 – 1.553) *p* 0.306

Pre-tax Household Income - >£100,000	1.00	1.00	1.00	1.00	1.00	1.00	1.00

Data presented as Odds Ratio (95% Confidence interval).

CHD=Coronary heart disease; HTN=Hypertension; T2DM=Type II diabetes mellitus; CKD=Chronic kidney disease.

*BMI not included in this analysis due to collinearity with obesity.

Supplementary Table 7: Logistic regression of risk of severe COVID-19 for model 3, presented as OR (95% CI)

	Odds Ratio (95% Confidence Interval)	*p* value
Coronary Heart Disease	1.146 (1.018 – 1.290)	0.025
Hypertension	1.666 (1.512 – 1.836)	<0.001
Type II Diabetes Mellitus	2.151 (1.941 – 2.385)	<0.001
Obesity	1.418 (1.298 – 1.548)	<0.001
Chronic Kidney Disease	2.454 (2.187 – 2.752)	<0.001
Depression	1.696 (1.514 – 1.900)	<0.001
Anxiety	1.806 (1.581 – 2.063)	<0.001
Age	1.027 (1.021 – 1.065)	<0.001
Townsend Deprivation Index	1.052 (1.038 – 1.065)	<0.001
Sex – Female	1.00	-
Sex - Male	1.650 (1.514 – 1.789)	<0.001
Ethnicity – White	1.00	-
Ethnicity – South Asian	1.870 (1.459 – 2.397)	<0.001
Ethnicity – Black	2.106 (1.673 – 2.652)	<0.001
Ethnicity – Other	1.262 (0.963 – 1.652)	0.092
Smoking Status – Never	1.00	-
Smoking Status – Previous	1.219 (1.113 – 1.334)	<0.001
Smoking Status - Current	1.502 (1.328 – 1.699)	<0.001
Qualifications – Degree	1.00	-
Qualifications – School	1.203 (1.073 – 1.348)	0.002
Qualifications – Professional	1.317 (1.145 – 1.515)	<0.001
Qualifications – None	1.536 (1.352 – 1.744)	<0.001
Pre-tax Household Income - <£18000	1.589 (1.197 – 2.110)	0.001
Pre-tax Household Income - £18000 to £30999	1.226 (0.925 – 1.624)	0.157
Pre-tax Household Income - £31000 to £51999	1.213 (0.916 – 1.606)	0.178
Pre-tax Household Income - £52000 to £100000	1.128 (0.845 – 1.507)	0.413
Pre-tax Household Income - >£100000	1.00	-

n = 396643.

Supplementary Table 8: Table displaying *p* values for the difference between odds ratios for risk of infection and severity per comorbidity, stratified by ethnicity.

Comparison ethnic groups		*p value* for difference between odds ratios	
		COVID-19 Infection	Severe COVID-19
Coronary Heart Disease	White and South Asian	0.030	<0.001
Hypertension	White and South Asian	<0.001	<0.001
	White and Black	0.0245	<0.001
Type II Diabetes	White and South Asian	0.0251	<0.001
	White and Black	0.323	<0.001
Obesity	White and South Asian	0.752	0.381
	White and South Asian	0.595	0.703
Chronic Kidney Disease	White and South Asian	0.6855	<0.001
	White and Black	<0.001	<0.001
Depression	White and South Asian	N/A*	0.433
	White and Black	N/A*	N/A*
Anxiety	White and South Asian	N/A*	0.391
	White and Black	N/A*	N/A*

*p-values for significant difference were only calculated for odds ratios that were significant in the original analyses (table 4; figure 3).
